# Heterogeneity of lipidomic profiles among lung cancer subtypes of patients

**DOI:** 10.1111/jcmm.13782

**Published:** 2018-07-12

**Authors:** Jiapei Lv, Danyan Gao, Yong Zhang, Duojiao Wu, Lihua Shen, Xiangdong Wang

**Affiliations:** ^1^ Zhongshan Hospital Institute of Clinical Science Shanghai Institute of Clinical Bioinformatics Fudan University Institute of Biomedical Science Fudan University Shanghai China

**Keywords:** biomarkers, lipidomics, lung cancer, subtypes

## Abstract

Lung cancer is a leading cause of cancer‐related deaths with an increasing incidence and poor prognoses. To further understand the regulatory mechanisms of lipidomic profiles in lung cancer subtypes, we measure the profiles of plasma lipidome between health and patients with lung cancer or among patients with squamous cell carcinomas, adenocarcinoma or small cell lung cancer and to correct lipidomic and genomic profiles of lipid‐associated enzymes and proteins by integrating the data of large‐scale genome screening. Our studies demonstrated that circulating levels of PS and lysoPS significantly increased, while lysoPE and PE decreased in patients with lung cancer. Our data indicate that lung cancer‐specific and subtype‐specific lipidomics in the circulation are important to understand mechanisms of systemic metabolisms and identify diagnostic biomarkers and therapeutic targets. The carbon atoms, dual bonds or isomerism in the lipid molecule may play important roles in lung cancer cell differentiations and development. This is the first try to integrate lipidomic data with lipid protein‐associated genomic expression among lung cancer subtypes as the part of clinical trans‐omics. We found that a large number of lipid protein‐associated genes significantly change among cancer subtypes, with correlations with altered species and spatial structures of lipid metabolites.

Lung cancer is a leading cause of cancer‐related deaths with an increasing incidence and poor prognoses, due to the lack of knowledge on heterogeneity complexity.^1^ Systems heterogeneity of lung cancer was described by integrating gene or protein expression, epigenetics, sequencing, transcription or interaction.^2^ Of those, there are a few studies on the heterogeneity of lipidomic profiles among lung diseases, which play an important role in the development, evolution, metabolism and mechanic function of lung. Marien et al^3^ investigated cancer and adjunct non‐malignant tissue contents of 91 phospholipid species in patients with small cell lung cancer (SCLC) and found that levels of SMs mainly reduced, while specific PIs elevated in cancer tissues. Reduced levels of PSs were correlated with some of elevated PE and PC species. Main changes were lipids with 40 or 42 carbon atoms in dual fatty acyl chains, with heterogeneity of phospholipids among cancer cells. Intratumour metabolic heterogeneity exists in cancer tissues, e.g renal cancer,^4^ contributing to the response of cancer cells to therapies. The human plasma lipidome is made up of thousands of ubiquitous lipid species to reflect systemic lipid metabolism and has the clinical relevance of diagnostics with the severity and risk of the disease. Although earlier studies implicated changes of lipid metabolism in tumour tissues,^3^, ^5^, ^6^ there is little known on systematic changes in lipidomic profiles in patients with lung cancer. The aim of our preliminary study is to explore the profiles of plasma lipidome between health and patients with lung cancer or among patients with squamous cell carcinomas (SCC), adenocarcinoma (ADC) or SCLC and to correct lipidomic and genomic profiles.

About 388 lipid molecules of plasma harvested from 8 health and 26 patients with SCC, ADC or SCLC were measured by MRM analysis performed with normal phase HPLC/MS. Patients with lung cancer as the first diagnosis and before any treatment were prospectively recruited at the initial clinic in Fudan University Zhongshan Hospital, without diabetes or other diseases. The study was approved by the Ethical Committee of Zhongshan Hospital. Total lipids were extracted from 200 μL plasma, performed with a modified method of Bligh & Dyer.^7^ Internal standard cocktails (Avanti Lipids Polar) were added at an amount of 10 μL to each sample, and lipid extracts were subjected to the normal‐phase silica liquid chromatography‐coupled triple‐quadrupole mass spectrometers (Qtrap^®^ 4000 and 6500, Sciex, Framingham, MA, USA). Both negative and positive ESI modes were used, the Q‐Trap was operated in the MRM mode, and different precursor/product ion pairs were scanned. Each experiment was repeated thrice. MRM data were processed with MultiQuant™ software (AB Sciex), and peak area of each pair was used for further quantification. Lung cancer specificity was identified to compare the pooled group of all subtypes with the health. The subtype specificity was defined as the level significantly higher or lower than that in the health (>twofold and *P* < 0.05), but not in other subtypes.

It was firstly reported that circulating levels of PS and lysoPS significantly increased, while lysoPE and PE decreased in patients with lung cancer. Our data demonstrated that the circulating levels of PE40s or lysoPE20s lipid species were significantly higher or lower in patients with lung cancer, respectively, of which about 28 lipid species co‐existed in all subtypes of lung cancer. It indicates that the systemic inflammation is in a high reactive condition, where cytokines may interact more with or cross local and systemic leucocytes or tumour cells.^8^ Lipidome comprehensive characterizations are dependent upon the number of carbon atoms, dual bonds or isomerism in the lipid molecule. For example, PS40:6, lysoPS22:6 or PS34:5 elevated >100‐fold, PS36:1 or 40:5, or lysoPS18:2 > 50, as well as PS34:1 or 34:2, lysoPS16:0 or 22:4 > 20, while d181So and lysoPE20:4(sn‐2) decreased >10‐fold, lysoPE20:3(sn‐2), 22:6(sn‐1) or 20:5(sn‐1) >5 and d171So, PS33:1, 33:2,35:4p or lysoPI22:0(sn‐1) >3. We further map the comprehensively lipidomic profiles and define subtype specificity of lung cancer, by comparing one subtype with healthy or other two subtypes. We noticed that 1 and 51 lipid species in SCC were significantly higher and lower, 34 and 6 in ADC or 5 and 4 in SCLC, respectively. Levels of PG or PI reduced mainly in SCC or ADC, while PS or PE elevated in ADC or SCLC, respectively (Table 1). Figure 1 demonstrates the heatmap of lipidomic profiles (Figure 1A), quantitative identification of main lipid elements, e.g PC, PS and PE (Figure 1B) and total levels of PC, PE, PS, SM and PI (Figure 1C) between health and lung cancer subtypes. The principal component analysis indicates the cover area (Figure 1D) and detailed distribution (Figure 1E) of lipidomic profiles in health and patients with various subtypes of lung cancer.

**Table 1:** Up‐ or down‐regulated type‐special lipids in serum over twofold in adenocarcinoma (ADC), squamous cell carcinoma (SCC) or small cell lung cancer (SCLC)

Lipid symbol	Fold	Lipid symbol	Fold
Up‐regulated type‐special lipids in SCC			
PE 34:1	2.413927591		
Up‐regulated type‐special lipids in ADC			
PS32:1	5.09765	lysoPS15:1	11.06539345
lysoPS20:2	17.472675	lysoPS20:1	18.84013333
PS30:1	19.00129722	PS38:6	19.63504722
lysoPS22:0	21.87011944	PS36:3	26.31176111
PS35:2	32.28302361	PS35:1	33.0320804
lysoPS16:0	35.35140556	PS34:0	37.26924167
PS34:3	50.05725958	PS38:5	56.80440113
PS38:2	59.53787133	lysoPS22:4	61.52068609
lysoPS20:3	63.70933056	PS34:2	77.12018239
lysoPS16:0	90.27303933	lysoPS18:2	94.63135557
PS36:0	100.2976925	PS40:4	103.1282059
PS38:4	110.1863088	PS36:1	130.7878593
PS40:5	141.3205679	lysoPS22:6	144.0307609
PS36:2	154.2687604	PS38:3	170.3411736
PS34:5	198.4490358	PS40:6	237.5144428
lysoPS18:1	271.9142996	lysoPS20:4	422.4742472
PS34:4	672.3756202	lysoPS18:0	772.2299397
Up‐regulated type‐special genes in SCLC			
PC 40:1	2.452430888	PC 40:4	3.035423914
PE 38:1	6.919780093	PE 42:7	8.224826755
PE 40:3	12.80649768		
Down‐regulated type‐special lipids in ADC			
PI 30:0	3.138308237	PI 33:0	2.973606913
PI 33:1e	2.756489035	PI 30:1	2.448825305
PI 33:2e	2.401033111	PI 41:6	2.089884861
Down‐regulated type‐special lipids in SCC			
lysoPI 18:2 (sn‐1)	3.174021852	lysoPI 17:0 (sn‐1)	2.686501872
PG36:2	2.599471242	PG35:2	2.599471242
PG35:3	2.582298818	PG35:1	2.573201161
PG34:0	2.571442278	PG39:7	2.57054263
PG37:3	2.568963178	PG35:4	2.560667263
PG34:1	2.544927869	PG36:0	2.540946865
PG40:1	2.533367656	PG37:6	2.519840973
PG36:1	2.517071946	PG33:1	2.509337261
PG41:6	2.508824969	lysoPG19:0	2.502973929
PG35:5	2.498092401	PG38:1	2.493572762
PG37:4	2.478698123	PG38:3	2.467495233
PG37:2	2.466940759	PG32:0	2.459511221
PG37:5	2.457177588	PG34:2	2.454550159
lysoPG22:4	2.445162623	lysoPG20:3	2.436735333
lysoPG22:0	2.414907832	PG40:4	2.412623442
PG36:4	2.411241381	lysoPG20:2	2.395150124
PG33:2	2.394734033	PG38:2	2.390172121
PG40:5	2.37513548	PG36:5	2.355426245
PG38:4	2.350017951	PG34:3	2.344849524
lysoPG20:4	2.321610499	PG31:0	2.318186638
lysoPG20:5	2.302249424	PG36:3	2.297141059
lysoPG20:0	2.296203929	lysoPG18:3	2.239909774
lysoPG17:0	2.238864462	PG40:8	2.236149498
PG38:6	2.231677958	PG38:5	2.21673939
PG39:3	2.163513382	PG40:6	2.162950362
PG40:7	2.139169557		
Down‐regulated type‐special lipids in SCLC			
lysoPC 20:5	3.679763534	d171S1P	2.622291711
PE 35:5p	2.377716405	Cer120	2.227294449

**Figure 1:**
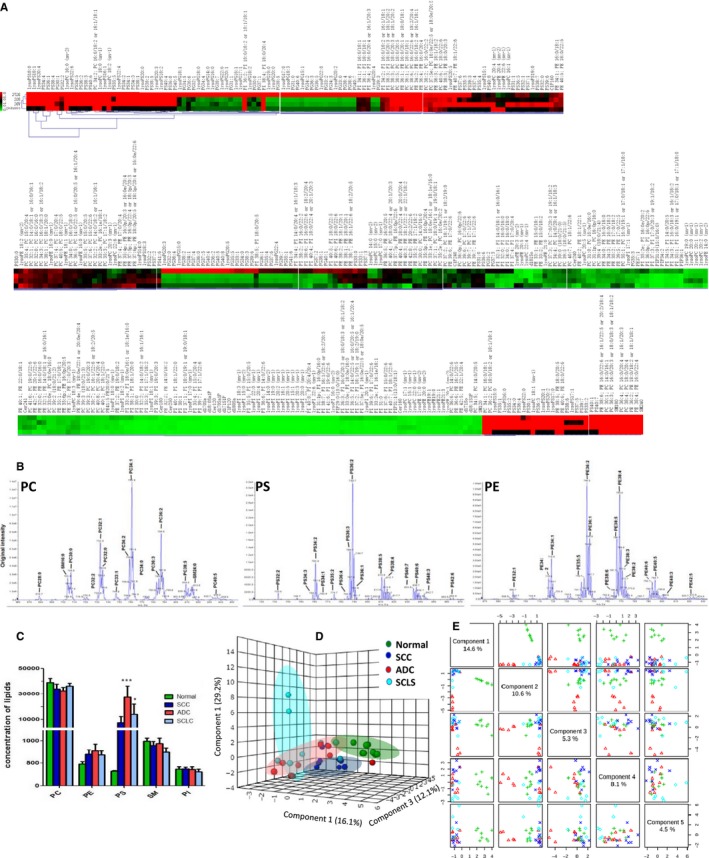
The Heatmap of Lipidomic Profiles (A) was Compared between the Health (as normal) and Patients with Squamous Cell Carcinomas (SCC), Adenocarcinoma (ADC) or Small Cell Lung Cancer (SCLC). The measurement quality of lipidomic profiles was shown by the precursor ion scan of phosphocholine (PC)‐, phosphatidylserine (PS)‐, phosphatidylethanolamine (PE)‐containing phospholipid species in plasma (B). The amounts (nmol lipid/mg DNA) of phospholipid head‐group classes, e.g PC, PE, PS, phosphatidylinositol (PI) or sphingomyelins (SM), were pooled and calculated among the health (as normal) or patients with SCC, ADC or SCLC, as presented in C (***<0.001). Three‐dimensional scatter plots of the principal component scores were illustrated in the validation set based on the weights from the discovery set (D). Component analysis of partial least squares‐discriminant analysis (PLS‐DA) by metabolism between the health (as normal) and patients with SCC, ADC or SCLC was scattered in E

Our results deliver the significant message that the heterogeneity of lipidomic profiles among lung cancer subtypes should be seriously considered and has the unique value of early diagnostics. For example, PI significantly increased with a decrease in PS in cancer tissues of patients with non‐small cell lung cancer,^3^ while there are no similar findings in plasma samples of patients with early lung cancer.^9^ Our present study demonstrated the elevation of PS in patients with lung cancer, as compared with healthy, rather than PI (Figure 1B). Altered PS species in the circulation can be valuable biomarkers to screen the risk population if those PS may be generated from lung cancer cells, or be an early lung cancer‐dependent pattern of lipid metabolism. The reduced amount of multi‐PS species within lung cancer cells can be due to the over‐consumed or over‐metabolized, to the over‐release of cytoplasm into the circulation or to the increased binding and activity of negatively charged lipids PS, PA and PG on lung cancer cell surfaces.^10^ The changed serum levels of phospholipids and their derivatives, e.g lysophosphatidylcholines and lysophosphatidylethanolamine, are considered as promising cancer markers.^9^, ^11^ On basis of previous finding that plasma level of the LPC reduced in patients with lung cancer and colorectal cancer,^12^ our results furthermore evidenced that lysoPG and lysoPI are lower in the plasma of patients with lung cancer than healthy individuals.

Lipid synthesis and its metabolites were suggested as a metabolic liability and potential biomarkers of lung cancer.^13^ Lipidome varied between the health and patients with lung cancer or among subtypes implies the existence of lipidomic heterogeneity, which may be caused by local and systemic cell metabolism and inflammation. To further understand the regulatory mechanisms of lipidomic profiles in lung cancer subtypes, we mined the genomic profiles of lipid‐associated enzymes and proteins from the global database integrated the data of large‐scale genome screening and other large mutation databases, as reported previously.^14^ We found that a large number of lipid protein‐associated genes significantly change among cancer subtypes, with correlations with altered species and spatial structures of lipid metabolites, like ACOT, ACSL, PKD gene families. Most of the long‐chain fatty acids (carbon atoms > 40) were up‐regulated in lung cancer. The over‐expression of fatty acid synthase (FASN) occurs in cancers, leading to the over‐production of endogenous fatty acids. Acyl‐CoA synthetases (ACSLs) enzymes, which convert free long‐chain fatty acids into fatty acyl‐CoA esters, also participate in the metabolic reprogramming of cancer cells. Recent study showed ACSL3 is the critical element for the oncogenic capacity of mutant KRAS in lung cancer.^15^

A number of genes and proteins are suggested as molecular biomarkers or therapeutic targets, which may contribute to the transit process from chronic obstructive pulmonary diseases to lung cancer.^16^ It is questioned whether lipid species or lipidomic profiles can be identified as stable biomarkers to monitor such transit process. It is also important to define the consistence and correlation of lipidomic profiles with genomic and proteomic ones. Our previous studies demonstrate that it is important and valuable to compare genomics and proteomics, while more important to integrate genomics and proteomics with clinical phenomes.^17^, ^18^, ^19^, ^20^ It will be a new challenge to merge or fuse lipidomics with clinical phenomics as the part of clinical trans‐omics.^21^ Thus, our data indicate that lung cancer‐specific and subtype‐specific lipidomics in the circulation are important to understand mechanisms of systemic metabolisms and identify diagnostic biomarkers and therapeutic targets. However, the roles of carbon atoms, dual bonds or isomerism in the lipid molecule in lung cancer cell differentiations should be further explored in future studies.

## CONFLICT OF INTEREST

The authors confirm that there are no conflicts of interest.
